# Reimagining cultural heritage conservation through VR, metaverse, and digital twins: An AI and blockchain-based framework

**DOI:** 10.1371/journal.pone.0335943

**Published:** 2025-11-03

**Authors:** Weiyi Zhang, Nooriati Taib, Mariati Taib

**Affiliations:** 1 School of Housing, Building and Planning, Universiti Sains Malaysia, Penang, Malaysia; 2 Jiangxi Institute of Fashion Technology, Nanchang, Jiangxi, China; IULM: Libera Universita di Lingue e Comunicazione, ITALY

## Abstract

Recent advances in artificial intelligence (AI), blockchain, virtual reality (VR), and digital twin technologies are transforming approaches to cultural heritage conservation. This study develops an integrated analytical framework that combines AI-driven modeling, interactive functionality, and blockchain/NFT authentication to examine both the direct and mediating effects of these technologies on heritage conservation effectiveness (HCE). Digital twins serve as a core component for simulating and managing heritage environments through dynamic, data-driven representations. An empirical analysis using Partial Least Squares Structural Equation Modeling (PLS-SEM) was conducted on 575 valid survey responses. The results indicate that blockchain/NFT authentication indirectly enhances heritage conservation effectiveness by improving digital authenticity. The study theoretically pioneers the integration of multiple digital technologies into a unified framework and empirically demonstrates the mediating roles of user immersive experience and digital authenticity. Practically, the findings offer actionable insights for advancing digital heritage conservation within metaverse environments and intelligent cultural ecosystems.

## 1. Introduction

In the digital era, cultural heritage conservation faces persistent challenges such as low data accuracy, unstable long-term storage, and limited public engagement. Conventional approaches often fail to capture the spatial complexity and cultural significance of heritage sites, resulting in information loss and diminished public awareness. Recognizing these shortcomings, international organizations such as UNESCO have emphasized the urgent need for digital transformation in heritage practices [1].

Emerging digital technologies have opened new avenues for advancing heritage conservation. Extended reality (XR) and metaverse platforms enable immersive visualization and participatory engagement, offering novel modes of heritage interpretation [2]. AI-driven modeling and building information modeling (BIM) techniques support high-fidelity 3D reconstruction and data integration, enhancing documentation accuracy and enabling adaptive reuse strategies [3]. Meanwhile, blockchain and non-fungible tokens (NFTs) provide secure and transparent frameworks for asset traceability and digital rights management in virtual environments [4]. Collectively, these technologies represent a paradigm shift in how heritage is documented, experienced, and governed. Yet, their combined impacts and underlying mechanisms remain insufficiently examined in current research.

Despite these advances, critical research gaps remain. Most studies examine these technologies in isolation, focusing on technical performance rather than their integrated effects within realistic heritage platforms [5]. Moreover, the mechanisms through which technological affordances influence conservation outcomes are rarely modeled. The roles of user immersive experience and digital authenticity as key determinants of trust, engagement, and perceived integrity in immersive environments remain underexplored [6]. In addition, empirical evidence linking these perceptual mechanisms to broader measures of heritage conservation effectiveness (HCE) is fragmented, limiting the development of sustainable, user-centered digital heritage systems.

Addressing these gaps is crucial for advancing both theory and practice. This study develops an integrated analytical framework that combines three technological dimensions: AI-driven modeling, interactive functionality, and blockchain/NFT authentication, to examine their direct and indirect effects on heritage conservation effectiveness. Using Partial Least Squares Structural Equation Modeling (PLS-SEM), the study further explores how immersive user experience and digital authenticity mediate the relationships between these technological affordances and heritage conservation effectiveness.

**Research question:** How do AI-driven modeling, interactive functionality, and blockchain/NFT jointly influence heritage conservation effectiveness, and to what extent are these relationships mediated by user immersive experience and digital authenticity?

This study makes three key contributions. First, it proposes a unified framework that systematically integrates these three technological dimensions in digital heritage conservation, bridging fragmented approaches. Second, it provides empirical evidence on the mediating roles of user immersive experience and digital authenticity, clarifying how technological affordances shape perceptions and heritage conservation effectiveness. Third, it advances both theoretical and practical understanding of how digital technologies can be designed to support sustainable, user-centered heritage platforms.

## 2. Literature review

### 2.1. Application of AI-driven modeling and automation technologies in cultural heritage conservation

AI-driven modeling and automation have become central to cultural heritage conservation, offering higher precision, efficiency, and scalability. Integrating AI with BIM facilitates accurate modeling and structural analysis. In parallel, 3D modeling and large-scale printing offer systematic approaches for restoring architectural elements with improved material efficiency and reduced reliance on scarce craftsmanship [7,8]. Advanced diagnostic technologies such as AI-assisted imaging and automated 3D scanning have enhanced the rigor of condition monitoring and preventive conservation, offering scalable solutions for heritage management [9–12].

Beyond technical optimization, these technologies have become integral to advancing broader heritage conservation objectives. AI-enabled documentation and analytical tools substantially enhance the fidelity of historical representations. By improving stratigraphic, material, and morphological accuracy, they reinforce the preservation of authenticity and ensure that restoration interventions remain faithful to original heritage characteristics [13]. This technological precision consolidates the evidentiary basis for conservation decision-making, bridging traditional craftsmanship with data-driven methodologies.

Moreover, AI-assisted platforms catalyze more inclusive and participatory heritage practices. Through virtual heritage environments and interactive educational applications, they democratize access to cultural knowledge and foster interpretive engagement across diverse communities [14,15]. In this regard, these tools facilitate a transition from expert-dominated preservation paradigms toward more collaborative, community-centered approaches, thereby broadening the social foundations of heritage stewardship.

From a long-term sustainability perspective, AI-driven systems enable predictive maintenance and data-informed conservation planning. Such approaches minimize resource consumption, support the adaptive reuse of heritage sites, and align conservation practices with global sustainability frameworks [16]. By integrating environmental, operational, and managerial considerations, they allow heritage institutions to reconcile preservation imperatives with practical implementation constraints [5]. Collectively, these technological functions support a more resilient, adaptive, and socially embedded framework for heritage conservation in the digital era. However, challenges remain in safeguarding authenticity, addressing ethical concerns, and managing cost constraints. These issues must be resolved to ensure responsible adoption and wider integration of these technologies into conservation practice.

### 2.2. Application of blockchain and NFTs technologies in digital cultural heritage conservation

Blockchain and non-fungible token (NFT) technologies have become key tools in digital cultural heritage conservation. They offer innovative approaches to ensure authenticity, provenance, and ownership security. The decentralized and tamper-resistant architecture of blockchain supports provenance tracking, property rights verification, and long-term preservation [17,18]. Recording ownership, restoration, and transaction histories on blockchain reduces the risks of data manipulation and unauthorized access [19].

NFTs provide verifiable digital identities for cultural objects, enabling ownership confirmation and value exchange in virtual environments such as the metaverse [20]. Recent studies highlight their role in ensuring authenticity and traceability while supporting transparent asset management and rights control [21,22]. Several museums and cultural institutions have begun to experiment with blockchain-based digital collections to enhance efficiency and security [23]. Smart contracts further streamline copyright and intellectual property management, reducing infringement risks and supporting sustainable value realization [24]. In response to these developments, UNESCO has advocated for regulating blockchain and NFT applications to ensure sustainable, equitable, and ethical heritage practices [25].

Despite these opportunities, critical barriers remain, including economic inefficiency [26], legal ambiguity [27], and governance risks [28], which limit the integration and scalability of blockchain technologies in heritage conservation. Limited digital literacy, particularly where NFTs are linked to speculative cryptocurrencies, constrains broader adoption [29]. Issues of data accessibility and user privacy introduce additional risks [30].

Tokenizing intangible cultural heritage (ICH), such as rituals, crafts, and oral traditions, raises significant ethical concerns. The transformation of Shu brocade patterns into NFTs restructures traditional value chains, raising questions about control and benefit distribution [31]. Similarly, the Quantum Temple project, while praised for empowering communities, embeds market logics into culturally sensitive domains [32]. Critics argue that NFT-driven “cryptoart” often reflects speculative economic models that risk commodifying culture and restricting equitable access [24,26,28]. These debates highlight the contested nature of NFT adoption and emphasize the need for culturally grounded ethical frameworks and inclusive governance models that prioritize community agency and values [21,24].

In summary, blockchain/NFT technologies offer promising opportunities for enhancing digital heritage preservation. However, their deployment demands careful attention to sustainability, legal frameworks, and cultural ethics. Future research should focus on co-designing blockchain systems with heritage communities, developing strong ethical guidelines, and establishing global standards to ensure responsible innovation. Continued engagement with real-world cases is essential to align technological applications with community values and to avoid cultural misappropriation.

### 2.3. Advances in VR, metaverse, and digital twin technologies for enhancing immersive experiences

Recent advances in virtual reality (VR), the metaverse, and digital twins (DT) technologies have reshaped immersive experience design for cultural heritage. Instead of functioning solely as visualization tools, these technologies now support interactive reconstruction, real-time monitoring, and participatory interpretation within heritage contexts [5]. VR enhances user immersion and educational engagement by enabling high-fidelity 3D reconstruction and interactive storytelling [33,34]. DT technologies complement these capabilities through continuous data integration and dynamic monitoring, supporting preventive conservation and ensuring accessibility through AR/VR applications [35].

The metaverse provides unique affordances for preserving, disseminating, and revitalizing cultural heritage. By combining VR and DT, it creates persistent, interactive environments where users can explore reconstructed sites beyond physical constraints. Digital twins support real-time monitoring and management, while immersive metaverse spaces enable educational storytelling and experiential learning [36,37]. These platforms also aid in restoring lost heritage through interactive 3D reconstructions, as in Babylon, Iraq [38]. They further enhance global accessibility by fostering inclusive participation and cultural exchange across geographic and economic boundaries [39,40].

Recent technological developments have addressed scalability and latency constraints in real-time heritage applications through the integration of DT, AI, blockchain, and advanced communication technologies [6]. These advances enhance data processing and responsiveness. Edge computing and reinforcement learning further optimize resource allocation in immersive environments. Meanwhile, brain–computer interface (BCI) applications provide cognitive and affective feedback that enables more adaptive and personalized experiences [41,42]. Together, these innovations create more intelligent, responsive, and user-centered heritage technologies.

Immersive technologies play a crucial role in shaping how users experience and trust cultural heritage. High-fidelity visualization, interactive storytelling, and participatory interpretation strengthen user immersive experience, deepening cognitive and emotional engagement [43,44]. Simultaneously, real-time data integration and blockchain-based traceability reinforce digital authenticity by ensuring accuracy, credibility, and transparent provenance [45,46]. Through these mechanisms, immersive technologies enhance heritage conservation effectiveness (HCE) by improving interpretive quality, fostering community trust, and supporting inclusive engagement. These mechanisms correspond to the key pathways outlined in the conceptual framework of this study.

Together, these technologies establish an intelligent and adaptive digital ecosystem for heritage conservation. They not only enhance immersion and personalization but also support evidence-based heritage management. Furthermore, they enable universal accessibility, providing the technical foundation for the theoretical model proposed in this study.

### 2.4. Integration of technological pathways and trends in engineering-oriented modeling

Recent advancements indicate a paradigm shift in digital cultural heritage preservation, moving from fragmented, tool-specific applications to comprehensive and intelligent system integration. The convergence of AI-driven modeling, VR and metaverse platforms, blockchain authentication, and digital twin technologies has enabled a transition toward data-informed, scalable, and engineering-oriented development frameworks. These integrated pathways enhance digitization fidelity, authenticity verification, and immersive user experiences. In addition, they enable modular and interoperable architectures that support long-term conservation strategies [2].

Technology-driven cultural heritage preservation has become a central focus of international research [3]. This engineering-oriented approach to heritage preservation research emphasizes systematic modeling, technical standardization, performance validation, and cross-platform interoperability. Instead of examining individual technologies in isolation, this paradigm promotes the development of integrated frameworks. These frameworks leverage AI algorithms to simulate environmental and behavioral dynamics [47], thereby enhancing predictive maintenance and user-centered interpretation within digital heritage environments [48,49]. Collectively, these unified frameworks automate data workflows, support empirical validation of preservation outcomes, and enhance replicability across diverse cultural contexts and technological infrastructures.

The field’s maturation necessitates future research to prioritize the development of integrated modeling frameworks that synergize algorithmic intelligence and engineering rigor. Empirical validation, system optimization, and real-world deployment will be key in transitioning cultural heritage conservation into an evidence-driven, sustainable, and technologically strong discipline.

## 3. Theoretical framework

To support the study’s technological framework and quantitative analysis, two interrelated models are proposed: the Technology Integration Framework (TIF) and the Digital Empowerment Model (DEM). Derived from a comprehensive review of digital heritage literature, these models serve complementary yet distinct roles within the overall conceptual framework.

TIF functions at the macro-structural level, integrating three core technological pathways: AI-driven modeling, interactive VR, and blockchain/NFT authentication. It highlights their synergistic contributions to digital heritage preservation and outlines the engineering logic underlying the structural model. This framework forms the conceptual foundation for the PLS-SEM approach used in this study.

In contrast, DEM operates at the meso-level, explaining the user-centered mechanisms through which these technologies exert influence. Specifically, it illustrates how AI-driven modeling and VR interaction enhance user immersive experience, and how blockchain/NFT reinforce digital authenticity. These two perceptual constructs serve as mediators, translating technological affordances into measurable heritage conservation effectiveness (HCE).

**Fig 1:**
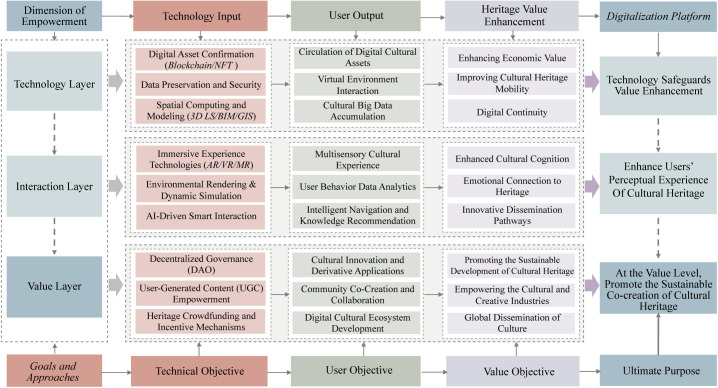
The conceptual model of the digital empowerment model (DEM). The diagram illustrates the application model of DEM in the context of cultural heritage conservation.

Together, TIF and DEM constitute a hierarchically nested and functionally complementary framework. TIF defines the system-level technological structure, while DEM reveals the experiential and cognitive pathways linking user engagement and perceived authenticity to heritage conservation effectiveness. Fig 1 presents the conceptual model. This dual-level integration ensures conceptual coherence between technological strategy and empirical analysis.

## 4. Methodology

This study employs Partial Least Squares Structural Equation Modeling (PLS-SEM) for quantitative modeling to empirically validate the proposed technical framework.

### 4.1. Research design

SmartPLS 4.0 was used to perform the structural equation modeling (SEM) analysis. The conceptual model was designed to evaluate the effects of three exogenous technology-oriented constructs: AI-driven modeling, interactive functionality, and blockchain/NFT authentication. These constructs were hypothesized to influence the mediating variables of user immersive experience and digital authenticity, which subsequently affect the outcome variable, heritage conservation effectiveness. This model specification enables a detailed assessment of both direct and indirect effects within the proposed theoretical framework.

### 4.2. Measurement model development

To ensure theoretical robustness and measurement validity of the structural model, all latent constructs were assessed using validated scales from prior literature, supplemented by a pre-test and expert evaluations.

User Immersive Experience (UIE): The measurement items for UIE were adapted from Slater’s [50] immersive experience scale, which evaluates sensory engagement, spatial presence, and emotional involvement in virtual environments. Example items include “I felt like I was really ‘inside’ the virtual environment” and “The experience felt real to me.” This scale has been widely applied in VR and metaverse research to assess immersion levels.

Digital Authenticity (DAU): DAU was assessed by evaluating the perceived integrity, reliability, and credibility of digital cultural assets. This scale was adapted from Kim and Kankanhalli’s [51] perceived information authenticity framework. Sample items include “The digital representation of the heritage was authentic” and “I found the digital assets trustworthy and credible.”

Heritage Conservation Effectiveness (HCE): Adapted from Paolanti et al. [52], the HCE scale evaluates the perceived efficacy of digital technologies in maintaining cultural significance, ensuring accuracy, and boosting public engagement. Sample items include “The project effectively preserved the cultural values of the heritage site” and “This digital initiative contributed to the long-term conservation of the heritage.”

Pre-test and Expert Validation: Before the large-scale survey, a pilot test involving 30 participants with VR and metaverse experience was conducted to refine item clarity and contextual relevance. Furthermore, three experts in digital heritage conservation and information systems reviewed the measurement items to ensure content validity. Minor revisions were made based on their feedback to enhance measurement reliability and alignment with the study context.

Latent variables were assessed using multiple items on a 5-point Likert scale, from 1 (strongly disagree) to 5 (strongly agree). The final measurement model incorporates established theoretical frameworks and empirical validation to ensure reliability and construct validity.

### 4.3. Measurement model construction

This study rigorously validates the impact mechanisms of AI-driven modeling, interactive functionality, and blockchain/NFT authentication on cultural heritage conservation by conceptualizing and defining the key constructs outlined in Table 1.

**Table 1:** Key research variables.

Construct	Measurement Items Example
AI-Driven Modeling (AIM)	AIM1-AIM4: AI precision, automation, model completeness
Interactive Functionality (IF)	IF1-IF4: Real-time interaction, sensory feedback
Blockchain/NFT Authentication (BC-NFT)	BC1-BC4: Ownership validation, traceability, security
User Immersive Experience (UIE)	UIE1-UIE4: Sense of presence, flow, engagement
Digital Authenticity (DAU)	DAU1-DAU4: Trust in data accuracy, perceived authenticity
Heritage Conservation Effectiveness (HCE)	HCE1-HCE5: Contribution to heritage preservation goals

The measurement model’s reliability and validity were rigorously evaluated through factor loadings, Composite Reliability (CR), Average Variance Extracted (AVE), and discriminant validity using the Heterotrait-Monotrait (HTMT) ratio. These assessments confirmed the robustness and measurement quality essential for structural equation modeling.

**Fig 2:**
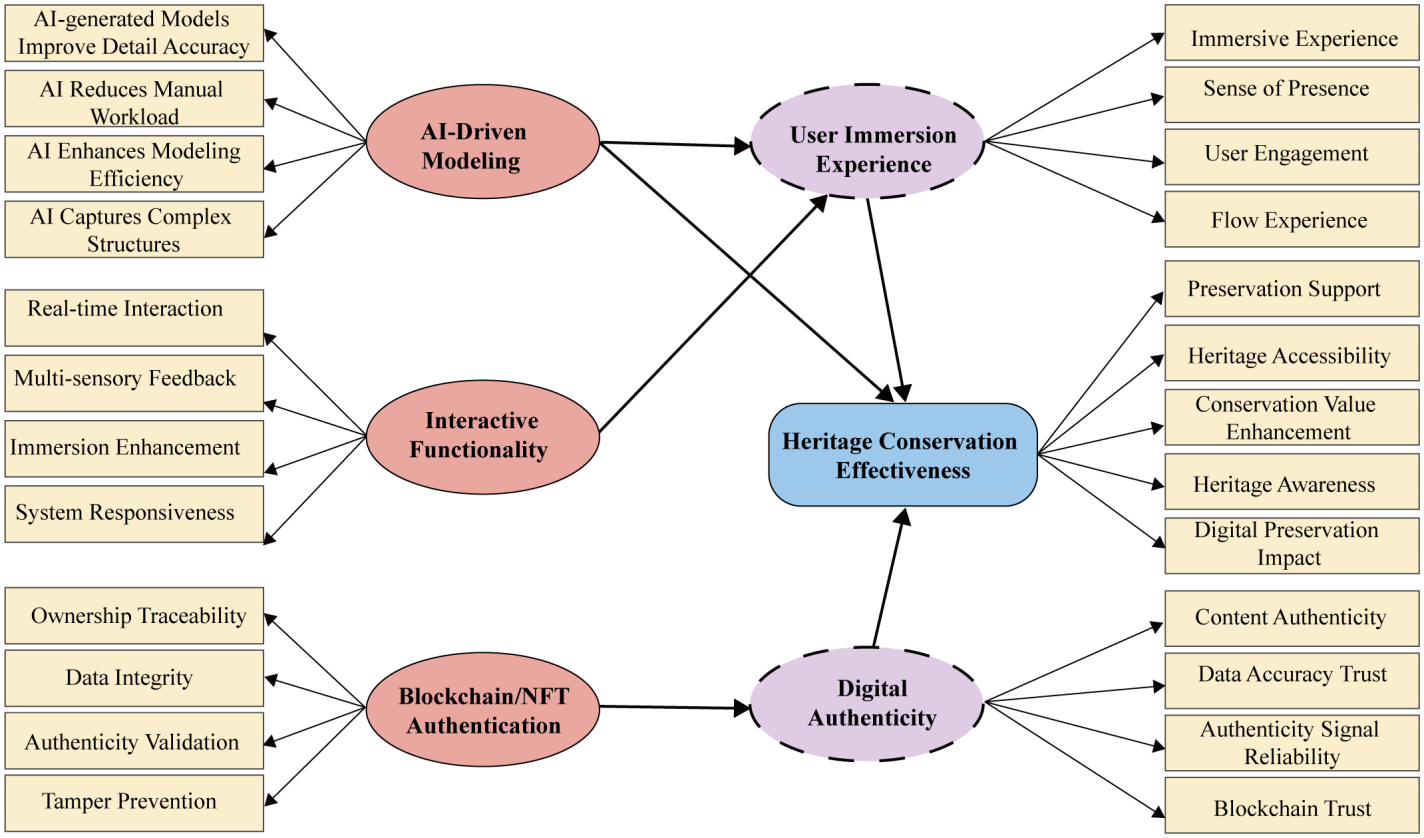
Hypothetical model of factors influencing the heritage conservation effectiveness.

The conceptual model presented in this study (Fig 2) systematically examines the impact of digital technologies on the effectiveness of cultural heritage conservation. It integrates six key variables: AI-driven modeling, interactive functionality, blockchain/NFT authentication, digital authenticity, user immersive experience, and heritage conservation effectiveness. Together, these variables form a comprehensive structural framework.

The study investigates the influence of three technological factors on user immersive experience and digital authenticity within AI-based and metaverse environments. These factors include AI-driven modeling, interactive functionality, and blockchain/NFT authentication. The model posits that the first two technological factors indirectly boost heritage conservation effectiveness (HCE) by enhancing user immersive experience. In contrast, blockchain/NFT authentication is expected to directly improve digital authenticity, which in turn positively affects heritage conservation effectiveness. The model also examines the mediating role of user immersive experience and explores how digital authenticity mediates the relationship between the technological factors and HCE.

The model demonstrates how AI-driven modeling, interactive functionality, and blockchain/NFT authentication can be integrated to enhance heritage conservation effectiveness. It leverages Virtual Reality (VR) and metaverse technologies to support this integration. In doing so, the model provides both theoretical and technical support for advancing digital cultural heritage preservation.

### 4.4. Research hypotheses

Based on the proposed research framework, the following hypotheses are formulated:

*H1*: AI-driven modeling positively influences user immersive experience.

*H2*: AI-driven modeling has a direct positive effect on heritage conservation effectiveness.

*H3*: Interactive functionality positively influences user immersive experience.

*H4*: User immersive experience positively contributes to enhancing heritage conservation effectiveness.

*H5*: Blockchain/NFT authentication positively influences digital authenticity.

*H6*: Digital authenticity positively influences heritage conservation effectiveness.

*H7*: User immersive experience mediates the relationship between AI-driven modeling and heritage conservation effectiveness.

*H8*: User immersive experience mediates the relationship between interactive functionality and heritage conservation effectiveness.

*H9*: Digital authenticity mediates the relationship between blockchain/NFT authentication and heritage conservation effectiveness.

### 4.5. Data collection methods

Participants were recruited via an anonymous online survey conducted from January 21 to February 20, 2025. The target population comprised residents of traditional dwellings in the Xidi and Hongcun regions. All responses were collected anonymously, ensuring no personally identifiable information was obtained. We collected 575 valid responses from participants with varying degrees of exposure to VR and metaverse technologies. Respondents were recruited via online platforms and offline cultural heritage centers to ensure a diverse and representative sample. A random sampling method was utilized to minimize potential.

This study was approved by the Research Ethics Committee of Jiangxi Institute of Fashion Technology, Jiangxi Province, China. The experiments are related to cultural heritage conservation and do not involve human or animal rights. Therefore, informed consent was not required, and a waiver of consent was granted by the Research Ethics Committee of Jiangxi Institute of Fashion Technology.

## 5. Findings

### 5.1. Descriptive statistics

Table 2 presents the demographic profile of the 575 respondents. Males constituted 57.7%, and females 42.3%. The predominant age group was 34–41 years (38.8%), followed by 26–33 years (22.8%) and 42–49 years (15.8%). Regarding education, 40.7% had a high/technical secondary school education, and 33.1% held a bachelor’s degree. Occupationally, 36.0% were in the enterprise/business sector, 32.5% were self-employed or freelancers, 13.0% worked in culture/tourism/museum, 9.7% in technology/digital media, and 8.7% in government, public institutions, or education. Overall, the sample distribution accurately reflects the target population’s characteristics.

**Table 2:** Demographic characteristics of the survey respondents (N = 575).

Sample Information		Frequency	Percentage (%)
Gender	Male	332	57.7
	Female	243	42.3
Age	(18, 25)	74	12.9
	(26, 33)	131	22.8
	(34, 41)	223	38.8
	(42, 49)	91	15.8
	(50, 60)	56	9.7
Educational Level	Elementary school and below	27	4.7
	Junior high school	94	16.3
	High/Technical secondary school	234	40.7
	Bachelor’s degree	190	33.1
	Master’s degree and above	30	5.2
Occupation	Technology/ Digital Media Industry	56	9.7
	Culture/ Tourism/ Museum Sector	75	13.0
	Government/ Public Institutions/ Education	50	8.7
	Enterprise/ Business Sector	207	36.0
	Self-employed/ Freelancer	187	32.5

### 5.2. Quantitative analysis results

#### 5.2.1. Reliability and validity.

This study evaluated data reliability and validity through the reflective measurement model. Reliability was determined using Cronbach’s alpha and composite reliability, while convergent validity was assessed via the Average Variance Extracted, as detailed in Table 3.

**Table 3:** Reliability and convergent validity of latent constructs.

Construct	Cronbach’s alpha	Composite reliability ($\rho_{A}$)	Composite reliability ($\rho_{C}$)	Average variance extracted (AVE)
**AIM**	0.707	0.744	0.816	0.531
**BC-NFT**	0.904	0.905	0.933	0.776
**DAU**	0.702	0.734	0.814	0.525
**HCE**	0.900	0.902	0.926	0.714
**IF**	0.900	0.901	0.930	0.770
**UIE**	0.811	0.836	0.876	0.641

**Note.** AI-driven modeling (AIM), Interactive functionality (IF), Blockchain/NFT authentication (BC-NFT), User immersive experience (UIE), Digital authenticity (DAU), Heritage conservation effectiveness (HCE), Composite reliability (CR), Average variance extracted (AVE).

(1) **Outer loadings**

Table 4 presents the outer loadings for all measurement items. The values range from 0.544 to 0.896, exceeding the recommended threshold of 0.5 and confirming acceptable indicator reliability. For AI-driven modeling (AIM), AIM2 has the lowest loading at 0.544, whereas AIM3 reaches 0.799. Although AIM2 shows a relatively low value, it remains within the acceptable range for exploratory studies. For blockchain/NFT authentication, loadings are high, between 0.855 and 0.896, indicating strong reliability. Digital authenticity (DAU) loadings range from 0.674 (DAU3) to 0.823 (DAU2). DAU3 has the lowest value but still exceeds the threshold. Interactive functionality (IF) demonstrates excellent reliability, with all items above 0.836 and IF4 reaching 0.896. User immersive experience (UIE) loadings vary from 0.671 (UIE2) to 0.891 (UIE3), indicating satisfactory reliability. All heritage conservation effectiveness (HCE) indicators exceed 0.812, with HCE4 reaching 0.881, which confirms strong measurement quality. Overall, the model demonstrates adequate indicator reliability. All items surpass the minimum loading of 0.5, and most exceed 0.7, supporting strong construct reliability.

**Table 4:** Outer loadings of measurement items.

No	Item Code	Outer Loading	No	Item Code	Outer Loading
1	AIM1	**0.792**	13	IF1	**0.836**
2	AIM2	**0.544**	14	IF2	**0.894**
3	AIM3	**0.799**	15	IF3	**0.883**
4	AIM4	**0.749**	16	IF4	**0.896**
5	BC-NFT1	**0.855**	17	UIE1	**0.837**
6	BC-NFT2	**0.896**	18	UIE2	**0.671**
7	BC-NFT3	**0.887**	19	UIE3	**0.891**
8	BC-NFT4	**0.884**	20	UIE4	**0.788**
9	DAU1	**0.686**	21	HCE1	**0.812**
10	DAU2	**0.823**	22	HCE2	**0.839**
11	DAU3	**0.674**	23	HCE3	**0.848**
12	DAU4	**0.706**	24	HCE4	**0.881**
			25	HCE5	**0.844**

**Note.** AI-driven modeling (AIM), Interactive functionality (IF), Blockchain/NFT authentication (BC-NFT), User immersive experience (UIE), Digital authenticity (DAU), Heritage conservation effectiveness (HCE).

(2) **Internal consistency**

This study evaluated the reliability of measurement scales by analyzing internal consistency using Cronbach’s alpha and Composite Reliability (CR), as detailed in Table 3. Cronbach’s alpha values ranged from 0.702 to 0.904, all above the recommended threshold of 0.70, indicating satisfactory internal consistency across constructs. Additionally, CR values ranged from 0.814 to 0.933, and other reliability metrics ranged from 0.734 to 0.905, all exceeding the suggested cutoff of 0.70 [53], thereby confirming the robustness and reliability of the measurement instruments.

(3) **Convergent validity**

Convergent validity evaluates construct validity by examining whether the indicators of a latent variable share substantial variance. This shared variance reflects their theoretical correlation. The Average Variance Extracted (AVE) was calculated to assess convergent validity, as shown in Table 3. All latent variables exhibited AVE values above 0.50, ranging from 0.525 (DAU) to 0.776 (BC-NFT). These values meet the threshold recommended by Hair et al. [53]. Overall, the results confirm that all latent variables demonstrate strong convergent validity.

(4) **Discriminant validity**

Discriminant validity evaluates the uniqueness of latent constructs by determining how distinct each construct is from the others in the model. This study used the Heterotrait–Monotrait Ratio (HTMT) as the evaluation criterion. HTMT is calculated as the ratio of correlations between indicators of different constructs (heterotrait) and those within the same construct (monotrait). This provides a reliable method for assessing discriminant validity. According to Hair et al. [53], HTMT values should not exceed 0.90, or 0.85 for more stringent evaluations. As shown in Table 5, all HTMT values in this study range from 0.542 to 0.862, which remain below the recommended thresholds. These findings strongly support the discriminant validity of all latent constructs.

**Table 5:** Discriminant validity based on Heterotrait–Monotrait Ratio (HTMT).

Construct	AIM	BC-NFT	DAU	HCE	IF	UIE
**AIM**						
**BC-NFT**	0.707					
**DAU**	0.745	0.604				
**HCE**	0.607	0.813	0.542			
**IF**	0.783	0.806	0.607	0.663		
**UIE**	0.840	0.862	0.657	0.747	0.832	

Note. AI-driven modeling (AIM), Interactive functionality (IF), Blockchain/NFT authentication (BC-NFT), User immersive experience (UIE), Digital authenticity (DAU), Heritage conservation effectiveness (HCE).

#### 5.2.2. Structural model and path coefficients.

The study employed a two-stage path analysis. The first stage assessed direct relationships, while the second evaluated mediating effects. The direct path analysis examined the impact of AI-driven modeling and interactive functionality on user immersive experience. It also investigated the influence of blockchain/NFT authentication on digital authenticity. Furthermore, the effects of user immersive experience and digital authenticity on heritage conservation effectiveness were evaluated.

The mediation analysis explored two key mechanisms. The first assessed the mediating role of user immersive experience in the relationships between AI-driven modeling and heritage conservation effectiveness, as well as between interactive functionality and heritage conservation effectiveness. The second examined how digital authenticity transmits the effect of blockchain/NFT authentication on heritage conservation effectiveness. This analytical framework aligns with the model’s hypothesized pathways. Results showed that most of the direct and indirect paths were statistically significant (p < 0.05), supporting the hypothesized relationships within the model.

(1) **Evaluation of collinearity**

Prior to hypothesis testing, multicollinearity was evaluated. The structural model examined collinearity between explanatory and predictor variables. Variance Inflation Factor (VIF) values were computed for each item to identify potential collinearity. All VIF values were below 5, indicating no significant multicollinearity, consistent with the threshold recommended by Hair et al. [53].

(2) **Path coefficients of structural model**

Structural relationships and standardized path coefficients (β) were assessed to evaluate construct effects. As shown in Table 6, the combined T-statistics and β values confirm the significance of the proposed model paths.

**Table 6:** Structural path coefficients and significance levels.

Structural Path	T-Statistics	β	P-Value
**AIM - > HCE**	2.810	0.127	0.005
**AIM - > UIE**	7.553	0.321	0.000
**BC-NFT - > DAU**	15.796	0.505	0.000
**DAU - > HCE**	2.858	0.116	0.004
**IF - > UIE**	12.444	0.515	0.000
**UIE - > HCE**	12.559	0.504	0.000
**BC-NFT - > DAU - > HCE**	2.658	0.059	0.008
**IF - > UIE - > HCE**	8.315	0.260	0.000
**AIM - > UIE - > HCE**	6.868	0.162	0.000

**Note.** AI-driven modeling (AIM), Interactive functionality (IF), Blockchain/NFT authentication (BC-NFT), User immersive experience (UIE), Digital authenticity (DAU), Heritage conservation effectiveness (HCE).

User immersive experience significantly enhances heritage conservation effectiveness (β = 0.504, p < 0.001). This indicates that user immersive experience substantially improves heritage conservation effectiveness. Among user immersive experience predictors, interactive functionality exerted the strongest effect (β = 0.515, p < 0.001), followed by AI-driven modeling (β = 0.321, p < 0.001). Blockchain/NFT authentication strongly influenced digital authenticity (β = 0.505, p < 0.001). In turn, digital authenticity positively affected heritage conservation effectiveness (β = 0.116, p = 0.004), confirming its mediating role. AI-driven modeling also showed a direct effect on heritage conservation effectiveness (β = 0.127, p = 0.005), further emphasizing its contribution to digital heritage conservation. Taken together, these findings highlight the central role of interactivity and authenticity in shaping effective digital heritage conservation.

**Fig 3:**
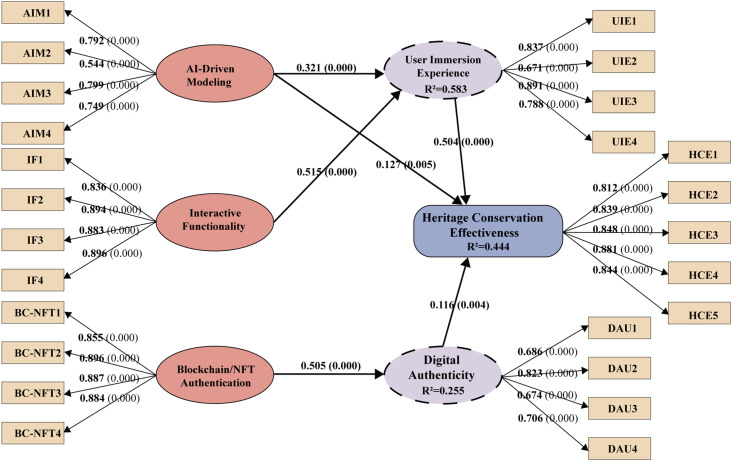
Path coefficients.

The mediation analysis revealed several significant indirect effects within the model. User immersive experience (UIE) mediated the relationships between AI-driven modeling (AIM) and heritage conservation effectiveness (HCE), as well as between interactive functionality (IF) and HCE. Likewise, digital authenticity (DAU) mediated the effect of blockchain/NFT authentication (BC-NFT) on HCE, emphasizing the roles of interactivity and authenticity in shaping heritage conservation effectiveness. The indirect path IF → UIE → HCE showed the strongest effect (β = 0.260, p < 0.001), highlighting the key role of immersion in translating interactivity into heritage conservation effectiveness. The path AIM → UIE → HCE also yielded a significant effect (β = 0.162, p < 0.001), indicating that user immersive experience partially mediates the impact of AI-driven modeling. The path BC-NFT → DAU → HCE showed a weaker yet significant effect (β = 0.059, p = 0.008), suggesting that blockchain/NFT authentication verification contributes to heritage conservation effectiveness. Overall, these findings highlight the mediating functions of user immersive experience and digital authenticity, thereby reinforcing the proposed theoretical framework. Detailed path coefficients are presented in Fig 3.

(3) $R^{\text{2}}$and $f^{\text{2}}$effect size

The coefficient of determination (R²) was assessed to gauge the model’s explanatory power and predictive accuracy for each endogenous construct. The results indicate an R² of 0.583 for user immersive experience, signifying moderate explanatory power. Similarly, heritage conservation effectiveness has an R² of 0.444, also indicating moderate explanatory power. In contrast, digital authenticity shows an R² of 0.255.

The f² effect sizes were calculated to evaluate the individual contributions of each predictor variable. According to Cohen’s [54] guidelines, values of 0.02, 0.15, and 0.35 denote small, medium, and large effects, respectively. The results reveal a large effect of interactive functionality on user immersive experience (f² = 0.370) and of blockchain/NFT authentication on digital authenticity (f² = 0.342). User immersive experience also exerted a medium effect on heritage conservation effectiveness (f² = 0.244), highlighting its key role in enhancing heritage conservation effectiveness. By contrast, negligible effect sizes were observed for the paths from AI-driven modeling (f² = 0.015) and digital authenticity (f² = 0.016) to heritage conservation effectiveness. These results indicate minimal contributions to the variance explained in heritage conservation effectiveness. Collectively, these findings confirm the explanatory power of the model and emphasize the substantial roles of IF and UIE within the structural framework.

## 6. Discussion

Based on the results above, this section discusses the theoretical and practical implications of the findings.

### 6.1. Direct effect analysis

Path analysis results from the PLS-SEM model show that all core hypotheses are significantly supported, confirming the model’s robustness and scientific validity.

H1 is supported. AI-driven modeling significantly enhances engineering value and user engagement. High-precision modeling increases the authenticity and detail of digital heritage restoration, providing a more immersive virtual experience. Consequently, AI-driven modeling is a crucial technological driver in digital heritage conservation. H2 validation confirms that AI-driven modeling directly boosts heritage conservation effectiveness through automation and precise reconstruction. These benefits maintain the integrity and accuracy of digital assets, facilitating optimized user immersive experience and interaction design in subsequent phases.

H3 is confirmed. Interactive functionality enhances user immersive experience, emphasizing the significance of interactive functionality in augmenting user perception and engagement. However, these features do not directly influence heritage conservation effectiveness (HCE). Their primary value is in optimizing user experience, which indirectly supports HCE at a perceptual level. H4 is supported. User immersive experience positively impacts HCE. This finding shows that subjective immersion and active engagement are key to effective digital heritage conservation.

H5 has been validated. Blockchain/NFT authentication bolsters digital authenticity through its tamper-proof and traceable characteristics. These features ensure the credibility and integrity of heritage data, rendering them indispensable to digital heritage conservation systems. H6 is supported. Heritage conservation effectiveness is bolstered by enhanced digital authenticity, highlighting the key role of authenticity perception in digital heritage conservation. This finding provides strong theoretical and empirical support for optimizing future digital heritage conservation strategies.

### 6.2. Mediation effect analysis

Mediation analysis reveals that AI-driven modeling, interactive functionality, and blockchain/NFT authentication significantly influence heritage conservation effectiveness via key mediators, validating the proposed technological pathways. Specifically, H7 is confirmed. AI-driven modeling directly enhances heritage conservation effectiveness and indirectly boosts it through user immersive experience. This indicates that AI-driven modeling is most effective when paired with strong user engagement and immersion.

H8 is confirmed, demonstrating that the influence of interactive functionality is entirely mediated by user immersive experience. This indicates that while interactive functionality enhances user experience, it does not directly affect heritage conservation effectiveness. H9 is validated, revealing that blockchain/NFT authentication enhances heritage conservation effectiveness by improving digital authenticity. This finding highlights the pivotal role of digital authenticity as both a technological foundation and an evaluative benchmark for heritage conservation effectiveness and digital heritage credibility.

Notably, the strength of mediation effects varies across pathways. The indirect effect of interactive functionality via user immersive experience (β = 0.260) is stronger than that of blockchain/NFT authentication via digital authenticity (β = 0.059). This suggests that experience-driven pathways exert a greater influence on heritage conservation effectiveness, whereas authenticity mechanisms play a comparatively smaller role.

Future research should employ indirect effect analysis to quantify the mediation strength of each pathway and elucidate dominant mechanisms. The model should also integrate external factors such as policy support, cultural identity, and economic feasibility to examine their moderating effects on technological pathways. Additionally, incorporating multi-group analysis and interaction modeling based on user demographics such as *gender*, *age*, and *experience* can reveal differences across user groups. This approach enhances understanding of experience-driven and digital authenticity, improves model applicability, and strengthens decision support, thereby providing a theoretical and empirical foundation for optimizing digital heritage conservation strategies.

### 6.3. Practical application scenarios and engineering validation

This study investigates the Huizhou digital restoration project and the Vatican metaverse Museum to assess the feasibility and practical value of AI-driven modeling, immersive technologies, and blockchain-based authentication in the conservation and dissemination of cultural heritage. These cases serve as practical validation of the proposed technological pathways and supplement the perception-based findings with real-world technical evidence.

#### Case 1: Huizhou digital restoration project.

This project focuses on the precise digital documentation and conservation of historical architecture by integrating advanced 3D surveying and information-modeling techniques [55]. An AI-driven analysis system supports automated detection of structural deterioration, thereby improving diagnostic accuracy and informing evidence-based restoration decisions [56]. Geographic information systems (GIS) facilitate spatial optimization and environmental analysis, improving monitoring and planning processes [57]. For cultural dissemination, the integration of VR, AR, and digital twins enhances public engagement and supports the digital transformation and global reach of Huizhou’s heritage [35,58]. This case exemplifies how AI and 3D modeling enhance conservation precision and broaden cultural accessibility.

#### Case 2: Vatican metaverse museum project.

This study confirms the efficacy of blockchain/NFT authentication for digitally authenticating heritage assets. Blockchain certification enhances the traceability and security of digital assets, effectively addressing the shortcomings of conventional digital archiving methods [21,59]. The VR-based metaverse exhibition transcends physical space constraints, significantly boosting user immersion and interaction rates by 20% to 60% [60]. Nonetheless, NFT-based authentication encounters challenges related to security, cultural value recognition, and market volatility, which pose concerns regarding its long-term sustainability [24].

These cases empirically support the study’s proposed technological pathways and mechanism design, validating the potential of AI-driven modeling, immersive technologies, and blockchain in heritage conservation. They also highlight key challenges such as authenticity, cost, and sustainability. These findings provide insights for refining models and advancing digital heritage conservation practices.

### 6.4. Conclusion and recommendations

This study establishes a systematic framework linking AI-driven modeling, interactive functionality, and blockchain/NFT authentication to heritage conservation effectiveness. AI-driven modeling and blockchain technologies have both direct and indirect effects, improving the perceived integrity and reliability of digital heritage preservation. Interactive functionality enhances user immersive experience, which mediates the relationship between technological engagement and conservation outcomes.

User immersive experience and digital authenticity act as key mediators translating technological innovation into measurable heritage value. Together, these mechanisms validate the Technology–Perception–Performance model and emphasize the central role of experience-driven authenticity in enhancing conservation effectiveness.

Overall, this research advances understanding of how digital technologies collectively strengthen authenticity assurance, user participation, and management precision. The proposed framework provides a replicable reference for integrating intelligent modeling and authentication into sustainable heritage conservation workflows. Future studies should extend this model across diverse cultural contexts and validate its long-term impacts through empirical and longitudinal analyses.

### 6.5. Policy and implications

This study presents a systematic framework and engineering pathway for integrating digital technologies into cultural heritage conservation. It elucidates the application potential and mechanisms of AI-driven modeling, VR interaction, and blockchain/NFT authentication.

The analysis of engineering challenges remains primarily qualitative, necessitating quantitative assessments and simulation models to enhance feasibility and practical value. AI-driven modeling in large-scale, high-precision applications encounters cumulative errors, with error rates in point cloud and image data ranging from 3% to 7%. These inaccuracies compromise the scientific validity of restoration and conservation decisions [61,62]. Future research should focus on developing error assessment models to quantify error sources and impacts, thereby enhancing engineering controllability and precision.

The high energy demand of blockchain/NFT authentication and immersive VR rendering presents critical sustainability challenges [18]. Applying life cycle assessment (LCA) methods within heritage digitization workflows can enhance carbon-efficiency evaluation and promote greener digital infrastructures. Future research should integrate energy monitoring and simulation to develop predictive VR energy models for sustainable design optimization.

At the policy level, a framework aligned with UNESCO’s AI Ethics Recommendation and national heritage strategies should be established to enhance the governance of AI, VR, and blockchain/NFT technologies, particularly in preventing counterfeits and managing value volatility within the cultural and creative sectors [22]. On the practical side, the proposed model demonstrates strong potential for applications in metaverse cultural tourism, digital museums, and rural revitalization. Specifically, AI-driven modeling enhances restoration precision, while blockchain ensures traceability and authenticity, thereby supporting sustainable cultural heritage conservation [4,23,63].

Future research should prioritize lightweight AI models, green blockchain technologies such as proof-of-stake or hybrid systems, and VR optimization. Moreover, integrating environmental and energy assessments is crucial for improving system scalability and ensuring long-term sustainability. In parallel, optimizing technological pathways and policy governance will be essential for advancing digital heritage conservation toward sustainable and practical applications. These efforts will provide both theoretical and empirical support for policymaking and industry.

### 6.6. Theoretical contributions and novelty

This study contributes significantly to the theoretical landscape of digital cultural heritage conservation. Firstly, it overcomes the limitations of single-technology approaches [21,22] by developing and validating an integrated model that synergizes AI-driven modeling, interactive functionality, and blockchain/NFT authentication. This model elucidates the synergistic effects of digital technologies and addresses a critical research gap in integration mechanisms. Secondly, the study introduces digital authenticity (DAU) as a mediating variable in a structural equation model. The results demonstrate that blockchain/NFT authentication indirectly enhances heritage conservation effectiveness by improving authenticity perception. This finding expands the theoretical application of digital authenticity in digital heritage conservation. Thirdly, the study identifies and verifies the mediating role of user immersive experience. The results show that AI-driven modeling and interactive functionality boost heritage conservation effectiveness through user immersion. This finding refines the perception mechanism framework in digital heritage conservation. Lastly, the research proposes the Technological Integration Framework (TIF) and the Digital Empowerment Model (DEM). Together, these models provide a scalable theoretical foundation for digital heritage conservation and metaverse applications. In summary, this study presents a comprehensive Technology, Perception, and Performance model. This model offers both theoretical and empirical insights for optimizing digital heritage conservation within metaverse contexts.

## 7. Limitations and future research

Despite revealing how AI-driven modeling, interactive functionality, and blockchain/NFT authentication enhance cultural heritage conservation, this study has several limitations requiring further investigation:

Data collection primarily utilized questionnaires, with samples skewed toward younger, highly educated individuals familiar with virtual heritage environments. This bias restricts the generalizability of the findings, particularly for the general public, older adults, and non-expert users. Future research should broaden sampling to encompass rural residents, cultural tourists, and museum visitors to enhance the model’s universality and applicability. Furthermore, integrating real-world projects, including museums, World Heritage sites, and intangible heritage bases, would facilitate empirical data collection and technical performance evaluation, thereby bolstering model validation and practical relevance.

Second, the current model primarily quantifies the relationships between technological pathways and user perception mechanisms but omits external variables such as policy environment, economic support, and social participation. This omission restricts the model’s adaptability and comprehensiveness within complex socio-technical systems. Future research should integrate factors such as policy frameworks, industry governance, and financial investment to develop a more comprehensive model with practical policy implications.

Third, the technical feasibility and environmental impact of emerging technologies such as blockchain, virtual reality (VR), and AI-driven modeling remain poorly quantified, hindering comprehensive evaluation. Future investigations are encouraged to integrate technical metrics such as *GPU energy consumption* and *blockchain processing efficiency* with standardized environmental assessment frameworks, including *life cycle analysis models*. This approach will enable rigorous quantification of resource utilization and ecological impacts across emerging technological pathways. This would enhance the model’s interpretability and predictive capacity. The current study provides early-stage empirical validation of conceptual relationships regarding users’ perceptions of integrated technologies. However, it did not directly assess the technical implementation and performance metrics of the AI, VR, and blockchain components. Subsequent research should develop and deploy operational prototypes to evaluate system stability, efficiency, and user-system interaction dynamics in real-world cultural heritage environments.

Finally, the cross-sectional design constrains the ability to observe dynamic changes in user experience, technological performance, and industrial benefits over time. Future research should employ longitudinal panel data and conduct empirical studies on representative heritage conservation projects. This approach would allow for a systematic evaluation of sustainability, development mechanisms, and technological performance throughout the lifecycle, offering strong empirical support for large-scale application and policy development.

## Supporting information

S1 File Relevant data for all analysis. (ZIP)
