# Supplementary material for: Reimagining cultural heritage conservation through VR, metaverse, and digital twins: An AI and blockchain-based framework
Source: PLoS One. 2025 Nov 3;20(11):e0335943. doi: 10.1371/journal.pone.0335943 (PMC12582480; doi:10.1371/journal.pone.0335943)
Supplement: S1 File — (ZIP) [file pone.0335943.s001.zip › S1 File. Relevant data for all analysis/S1_Questionnaire_and_Variables.pdf]

**Questionnaire and Variables**

| Latent variables | Observation Variables | Variable Valuations |
|---|---|---|
| Sample Characteristics (SC) | SC1: Gender | Male = 1; Female = 2 |
| | SC2: Age | (18, 25)=1; (26, 33)=2; (34, 41)=3; (42, 49)=4; (50, 60)=5 |
| | SC3: Educational Level | Elementary school and below = 1; Junior high school= 2; High /Technical secondary school= 3; Bachelor's degree = 4; Master’s degree and above= 5 |
| | SC4: Occupation | Technology / Digital Media Industry= 1; Culture / Tourism / Museum Sector= 2; Government / Public Institutions / Education= 3; Enterprise / Business Sector = 4; Self-employed / Freelancer= 5 |
| AI-Driven Modeling (AIM) | AIM1: AI-generated models improve detail accuracy | Strongly Disagree= 1; Disagree= 2; Neutral / Neither Agree nor Disagree = 3; Agree = 4; Strongly Agree = 5 |
| | AIM2: AI reduces manual workload | Strongly Disagree= 1; Disagree= 2; Neutral / Neither Agree nor Disagree = 3; Agree = 4; Strongly Agree = 5 |
| | AIM3: AI enhances modeling efficiency | Strongly Disagree= 1; Disagree= 2; Neutral / Neither Agree nor Disagree = 3; Agree = 4; Strongly Agree = 5 |
| | AIM4: AI captures complex structures | Strongly Disagree= 1; Disagree= 2; Neutral / Neither Agree nor Disagree = 3; Agree = 4; Strongly Agree = 5 |

| | | |
|---|---|---|
| Interactive Functionality (IF) | IF1:Real-time Interaction | Strongly Disagree= 1; Disagree= 2; Neutral / Neither Agree nor Disagree = 3; Agree = 4; Strongly Agree = 5 |
| | IF2:Multi-sensory Feedback | Strongly Disagree= 1; Disagree= 2; Neutral / Neither Agree nor Disagree = 3; Agree = 4; Strongly Agree = 5 |
| | IF3:Immersion Enhancement | Strongly Disagree= 1; Disagree= 2; Neutral / Neither Agree nor Disagree = 3; Agree = 4; Strongly Agree = 5 |
| | IF4:System Responsiveness | Strongly Disagree= 1; Disagree= 2; Neutral / Neither Agree nor Disagree = 3; Agree = 4; Strongly Agree = 5 |
| Blockchain/NFT Authentication(BC-NFT) | BC-NFT1: Ownership of digital heritage is traceable | Strongly Disagree= 1; Disagree= 2; Neutral / Neither Agree nor Disagree = 3; Agree = 4; Strongly Agree = 5 |
| | BC-NFT2: Blockchain ensures data integrity | Strongly Disagree= 1; Disagree= 2; Neutral / Neither Agree nor Disagree = 3; Agree = 4; Strongly Agree = 5 |
| | BC-NFT3: NFTs validate authenticity | Strongly Disagree= 1; Disagree= 2; Neutral / Neither Agree nor Disagree = 3; Agree = 4; Strongly Agree = 5 |
| | BC-NFT4: Blockchain prevents tampering | Strongly Disagree= 1; Disagree= 2; Neutral / Neither Agree nor Disagree = 3; Agree = 4; Strongly Agree = 5 |
| User Immersive Experience (UIE) | UIE1: The experience feels highly immersive | Strongly Disagree= 1; Disagree= 2; Neutral / Neither Agree nor Disagree = 3; Agree = 4; Strongly Agree = 5 |
| | UIE2: I feel a sense of presence | Strongly Disagree= 1; Disagree= 2; Neutral / Neither Agree nor Disagree = 3; Agree = 4; Strongly Agree = 5 |
| | UIE3: The system engages me fully | Strongly Disagree= 1; Disagree= 2; Neutral / Neither Agree nor Disagree = 3; Agree = 4; Strongly Agree = 5 |

| | | |
|---|---|---|
| | UIE4: The flow experience is strong | Strongly Disagree= 1; Disagree= 2; Neutral / Neither Agree nor Disagree = 3; Agree = 4; Strongly Agree = 5 |
| Digital Authenticity (DAU) | DAU1: Digital content feels authentic | Strongly Disagree= 1; Disagree= 2; Neutral / Neither Agree nor Disagree = 3; Agree = 4; Strongly Agree = 5 |
| | DAU2: I trust the accuracy of the digital heritage | Strongly Disagree= 1; Disagree= 2; Neutral / Neither Agree nor Disagree = 3; Agree = 4; Strongly Agree = 5 |
| | DAU3: System provides reliable authenticity signals | Strongly Disagree= 1; Disagree= 2; Neutral / Neither Agree nor Disagree = 3; Agree = 4; Strongly Agree = 5 |
| | DAU4: Blockchain increases my trust | Strongly Disagree= 1; Disagree= 2; Neutral / Neither Agree nor Disagree = 3; Agree = 4; Strongly Agree = 5 |
| Heritage Conservation Effectiveness (HCE) | HCE1: System supports preservation goals | Strongly Disagree= 1; Disagree= 2; Neutral / Neither Agree nor Disagree = 3; Agree = 4; Strongly Agree = 5 |
| | HCE2: Enhances accessibility to heritage | Strongly Disagree= 1; Disagree= 2; Neutral / Neither Agree nor Disagree = 3; Agree = 4; Strongly Agree = 5 |
| | HCE3: Increases long-term conservation value | Strongly Disagree= 1; Disagree= 2; Neutral / Neither Agree nor Disagree = 3; Agree = 4; Strongly Agree = 5 |
| | HCE4: Strengthens user awareness of heritage | Strongly Disagree= 1; Disagree= 2; Neutral / Neither Agree nor Disagree = 3; Agree = 4; Strongly Agree = 5 |
| | HCE5: Digital Preservation Impact | Strongly Disagree= 1; Disagree= 2; Neutral / Neither Agree nor Disagree = 3; Agree = 4; Strongly Agree = 5 |
