# Supplementary figures and images for: Reimagining cultural heritage conservation through VR, metaverse, and digital twins: An AI and blockchain-based framework
Source: PLoS One. 2025 Nov 3;20(11):e0335943. doi: 10.1371/journal.pone.0335943 (PMC12582480; doi:10.1371/journal.pone.0335943)

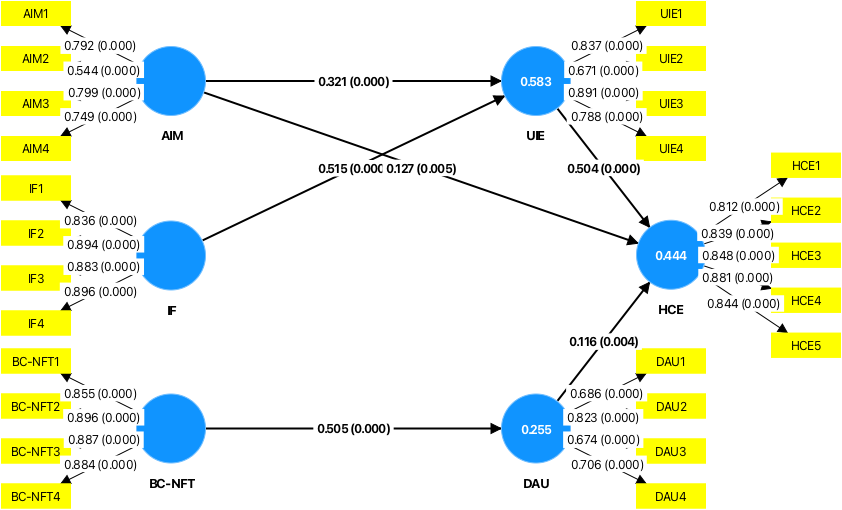

Supplement: S1 File — (ZIP) [file pone.0335943.s001.zip › S1 File. Relevant data for all analysis/S1_PLS_SEM_Model_Figure.png]
